# A novel scheme to design the filter for CT reconstruction using FBP algorithm

**DOI:** 10.1186/1475-925X-12-50

**Published:** 2013-06-01

**Authors:** Hongli Shi, Shuqian Luo

**Affiliations:** 1 School of Biomedical Engineering, Capital Medical University of China, Beijing 100069, China

**Keywords:** Filtered Back-Projection (FBP) algorithm, Computerized tomographic (CT) imaging, Reconstruction, Projection, Optimization

## Abstract

**Background:**

The Filtered Back-Projection (FBP) algorithm is the most important technique for computerized tomographic (CT) imaging, in which the *ramp* filter plays a key role. FBP algorithm had been derived using the continuous system model. However, it has to be discretized in practical applications, which necessarily produces distortion in the reconstructed images.

**Methods:**

A novel scheme is proposed to design the filters to substitute the standard *ramp* filter to improve the reconstruction performance for parallel beam tomography. The design scheme is presented under the discrete image model and discrete projection environment. The designs are achieved by constrained optimization procedures. The designed filter can be regarded as the optimal filter for the corresponding parameters in some ways.

**Results:**

Some filters under given parameters (such as image size and scanning angles) have been designed. The performance evaluation of CT reconstruction shows that the designed filters are better than the ramp filter in term of some general criteria.

**Conclusions:**

The 2-D or 3-D FBP algorithms for fan beam tomography used in most CT systems, are obtained by modifying the FBP algorithm for parallel beam tomography. Therefore, the designed filters can be used for fan beam tomography and have potential applications in practical CT systems.

## Background

**Figure 1:**
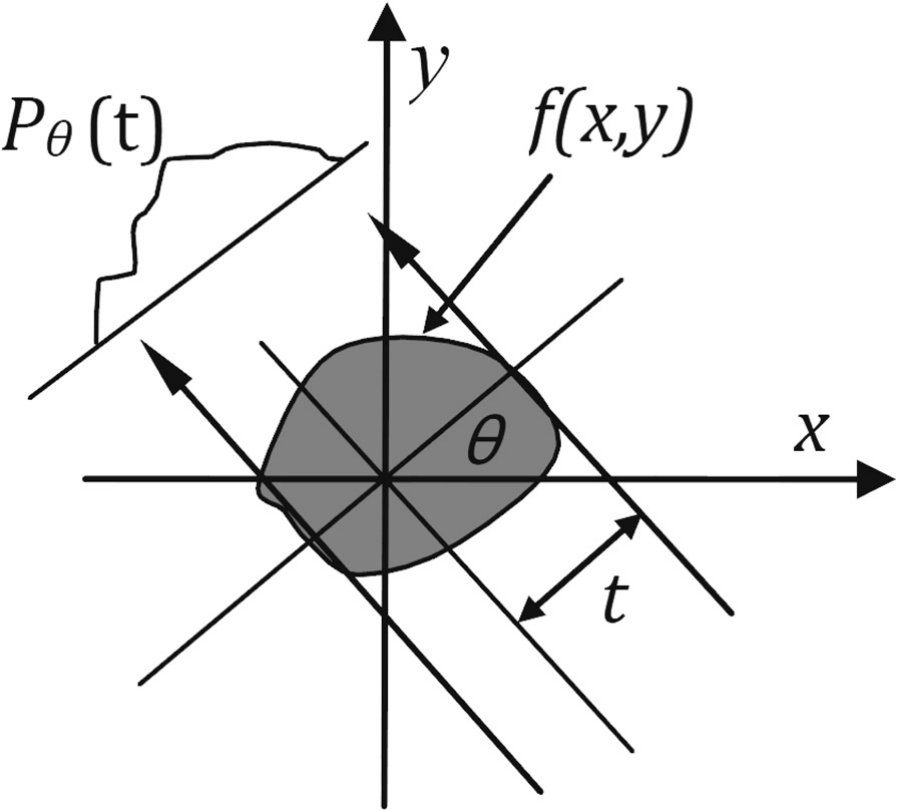
**Parallel Projection: an object*f(x,y)* and its projection*P***_***θ***_***(t)* are shown from the angle*θ*.**

X-ray CT imaging is a procedure to get internal information of an unknown object, such as biological tissue, from the projection data collected by illuminating the object from many different directions using X-ray. The object can be represented by its distribution of X-ray attenuation coefficient. When a parallel beam of X-rays propagates through the object, the total attenuation of the beam can be expressed by a line integral, which is the well-known Radon transform [1,2]

$$p_{\theta }(t)=\int_{-\infty }^{\infty }f(x,y)\delta (x\cos\theta +y\sin\theta -t)\text{dxdy},$$

where *f*(*x*,*y*) denotes the object (or its distribution of X-ray attenuation coefficient); *p*_*θ*_(*t*) denotes the projection data when the scanning angle is *θ* and the distance between the projection line and the origin is *t*; *δ*(·) denotes the Dirac delta function or the impulse function; *x* cos*θ*+*y* sin*θ*−*t* represents a projection line of X-rays, as shown in Figure 1.

The reconstruction procedure is very important for CT imaging. The properties of the final reconstructed image heavily depends upon the reconstruction algorithm used. Many algorithms have been proposed. They can be roughly divided into three categories: 1) analytical schemes, 2) algebraic reconstruction technique (ART) and 3) statistical iterative reconstruction (SIR) schemes. Some of the ART and SIR algorithms have become hot topics in CT reconstruction research, however, these categories suffer from their heavy calculation burden, poor convergence speed and other drawbacks [3-7]. For example, the SIR algorithms lack an efficient stop criterion, and ART algorithms are sensitive to noise in the projection data. Both categories of algorithms can only been used in a few special fields. The analytical schemes are much simpler and faster. Of these FBP algorithm is the most important one [1,8-11]. FBP algorithm and its modified versions for 2-D and 3-D projection reconstruction, such as FDK (Feldkamp-Davis-Kress) algorithm, have been used in almost all the fields of straight ray tomography, such as X-ray CT and PET (Positron Emission Tomography) [12-14]. The projections can be classified into two types: parallel and fan beam projection. Since the FBP algorithm for fan beam tomography is usually obtained by modifying that for parallel beam tomography, only the latter is studied in this paper. The derivation of FBP algorithm for parallel beam tomography is rather simple and straightforward. First, the Fourier slice theorem links 1-D Fourier transform (FT) of the projection data collected at angle *θ*, *S*_*θ*_(*ω*), with 2-D FT at the frequency samples (*ω* cos*θ*,*ω* sin*θ*), *F*(*ω* cos*θ*,*ω* sin*θ*). That is

(1) $$\begin{array}{ll} S_{\theta }(\omega ) & =\int_{-\infty }^{\infty }P_{\theta }(t)\exp(-i2\pi \omega t)dt\\ & =\int_{-\infty }^{\infty }\int_{-\infty }^{\infty }f(x,y)\exp\left(-i2\pi \omega \left(x\cos\theta +y\sin\theta \right)\right)dxdy\\ & =F(\omega \;\cos\;\theta ,\omega \;\sin\theta ). \end{array}$$

Then the unknown *f*(*x*,*y*) can be reconstructed by the inverse Fourier transform (IFT) or the dual Radon transform as following

$$\begin{array}{ll} \hat{f}(x,y) & =\int_{0}^{\pi }\int_{-\infty }^{\infty }F(\omega \cos\theta ,\omega \sin\theta )|\omega |\exp(i2\pi \omega (x\cos\theta +y\sin\theta ))d\omega d\theta \\ & =\int_{0}^{\pi }\int_{-\infty }^{\infty }S_{\theta }(\omega )|\omega |\;\exp(i2\pi \;\omega t)d\omega d\theta , \end{array}$$

where $\hat{f}(x,y)$ denotes the reconstructed image; *t*=*x* cos*θ*+*y* sin*θ*; |*ω*| is known as “ramp” filter in the frequency domain. It is well-known that $\hat{f}(x,y)$ will be identical with *f*(*x*,*y*) almost everywhere according to the properties of FT and IFT.

In practice, the projection data and reconstructed images have to be discretized to record, calculate and display. For the discrete projection data, $P_{\theta_{j}}(l),l\in \;[-\frac{N}{2},\cdots \;,\frac{N}{2}]$, the discrete Fourier transform (DFT) and inverse DFT (IDFT) are employed to approximate (continuous) FT and IFT, respectively. They are

(2) $$\begin{array}{l} S_{\theta_{j}}(\omega )\approx S_{\theta_{j}}(k)=\overset{\frac{N}{2}-1}{\underset{l=-\frac{N}{2}}{\sum }}P_{\theta_{j}}(l)\exp(-i2\pi \frac{\text{lk}}{L}),\;k\in \;[-\frac{L}{2},\cdots \;,\frac{L}{2}],\\ \hat{f}(n,m)\approx \frac{\pi }{K}\overset{K}{\underset{j=1}{\sum }}Q_{\theta_{j}}(l),\\ Q_{\theta_{j}}(l)=\frac{1}{L+1}\overset{\frac{L}{2}}{\underset{k=-\frac{L}{2}}{\sum }}S_{\theta_{j}}(k)\left|\frac{k}{L}\right|\exp\left(i2\pi \frac{\lfloor n\cos\theta_{j}+m\sin\theta_{j}\rceil k}{L}\right), \end{array}$$

where *N* is a positive even integer denotes the number of projection data; *L* is an even integer that is equal to or larger than the maximum number of the discrete projection data at all directions; ⌊*x*⌉ denotes the nearest integer of *x*; *θ*_*j*_,*j*∈ [ 1,⋯,*K*], denote the discretized scanning angle, and *K* is the number of the scanning angles. The discretized ramp filter, $\left|\frac{k}{L}\right|$, is named as the reconstruction filter in this paper.

For the continuous systems, Radon and inverse Radon transforms are solid and perfect in the mathematics principle [8-10]. However, it necessarily produces non-negligible degradation when the projection data are discrete (finite) and Radon and inverse Radon transforms have to be discretized in calculation. Many scholars have studied this problem. In [15], a multilevel back-projection method had been presented to improve the computational speed. The point-spread-function (PSF) convolution techniques had been proposed to reduce blurring. By those approaches the image quality was similar with or superior to that using the standard FBP technique. In [16], the spline interpolation and ramp filtering had been combined to improve the standard FBP algorithm, by which the image quality could also be improved somewhat.

The question can be summarized as how to reduce the degradation caused by the discretizing process. Since the degradation cannot be removed completely, the question can also be simplified as how to design the optimal reconstruction filter for the discrete inverse Radon transform. In this paper, we try to solve this question in a quite different way. First, the discrete image model and discrete projection model are employed in simulating the scanning procedure. The DFT of projection data is regarded as a special 2-D DFT of the original image using non-uniform frequency sampling. Then, the reconstructed image is regarded as a convolution of the original image and a particular kernel. The kernel is constructed by 2-D DFT, IDFT and the reconstruction filter to be designed. If the kernel become a 2-D Dirac delta function, the reconstructed image will be identical with the original image. So, the optimal reconstruction filter can be obtained by an optimization procedure that make the kernel approach the 2-D Dirac delta function as near as possible.

In order to make the idea behind the design scheme clear, a similar question for 1-D signal is proposed at first, and then it is extended to 2-D situations to solve the corresponding question in CT reconstruction.

## Methods

### The reconstruction filter for 1-D signal

Suppose a 1-D real signal *x*(*n*),*n*∈ [ 0,⋯,*N*−1], where *N* is the length of the signal. A special form of DFT is defined as following

(3) $$X(k)=\overset{N-1}{\underset{n=0}{\sum }}x(n)\exp\left(-i\theta_{k}n\right),\;\;k\in \;[\;0,\cdots \;,N-1],$$

where *θ*_*k*_ denote *N* angles on the region [ 0,2*π*] correspond to *N* frequency samples. Similarly, the IDFT is defined as following

(4) $$\hat{x}(n)=\frac{1}{N}\overset{N-1}{\underset{k=0}{\sum }}X(k)\exp\left(i\theta_{k}n\right),\;\;n\in \;[\;0,\cdots \;,N-1],$$

where $\hat{x}(n)$ denotes the reconstructed signal.If $\theta_{k}=\frac{2k\pi }{N}$, (3) and (4) become the standard DFT and IDFT, respectively. It is well-known the original signal *x*(*n*) can be perfectly reconstructed when it is transformed into the frequency domain and then transformed back using the standard DFT and IDFT. That is

$$\begin{array}{ll} \hat{x}(n) & =\frac{1}{N}\sum_{k=0}^{N-1}X(k)\exp\left(i2\pi kn/N\right)\\ & =\frac{1}{N}\sum_{m=0}^{N-1}x(m)\sum_{k=0}^{N-1}\exp\left(i2\pi k(n-m)/N\right)=x(n). \end{array}$$

**Figure 2:**
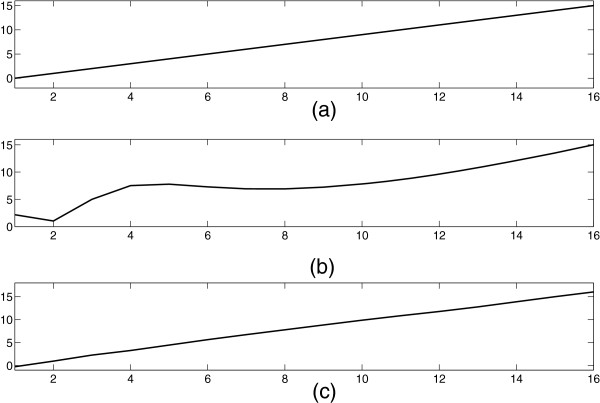
**The original signal and its different reconstructed versions.(a)** The original signal, **(b)** the reconstructed signal after DFT and IDFT using *θ*_*k*_=(2*kπ*/*N*)^0.8^, **(c)** the reconstructed signal after the identical DFT, IDFT and a filtering process using the designed filter between DFT and IDFT.

However, it may happen $\theta_{k}\neq \frac{2k\pi }{N}$ in some special situations, i.e., the frequency samples do not distribute uniformly in the frequency region [ 0,2*π*]. When $\theta_{k}\neq \frac{2k\pi }{N}$, the original signal cannot be reconstructed exactly. That is say, the nonuniformity of frequency sampling will produce distortion in the reconstructed signal. A simple example is presented to show what will happen when the frequency samples are non-uniform. Suppose the original signal is *x*=*n*,*n*∈ [ 0,1,⋯,15], which is plotted in Figure 2 (a). It is transformed into the frequency domain and then transformed into the time domain using the frequency samples $\theta_{k}={\left(\frac{2k\pi }{N}\right)}^{0.8}$, *k*∈ [ 0,1,⋯,15]. The reconstructed signal is plotted in Figure 2 (b), which is quite different from the original signal.

Generally, the original signal can still be reconstructed exactly from *X*(*k*) even in such a situation according to mathematics analysis. However, it may bring in heavy computational burden. For example, it may involve the calculation of the inverse or Moore-Penrose pseudo inverse of a large matrix. In order to avoid such a problem, we propose an alternative approach. At first, the reconstructed signal is expressed as following

(5) $$\begin{array}{ll} \hat{x}(n) & =\frac{1}{N}\sum_{k=0}^{N-1}X(k)\;\exp\;\left(i\theta_{k}n\right)\;=\;\frac{1}{N}\sum_{k=0}^{N-1}\sum_{m=0}^{N-1}x(m)\;\exp\;(-i\theta_{k}m)\;\exp\;(i\theta_{k}n)\\ & =\frac{1}{N}\sum_{m=0}^{N-1}x(m)\sum_{k=0}^{N-1}\;\exp\;\left(i\theta_{k}(n-m)\right)=\sum_{m=0}^{N-1}x(m)h(n-m), \end{array}$$

where $h(n-m)=\frac{1}{N}\overset{N-1}{\underset{k=0}{\sum }}e^{i\theta_{k}(n-m)}$. The reconstructed signal can further be expressed as the periodic (or circular) convolution of **h** and **x**,

(6) $$\hat{x}=h⊙x,$$

where ⊙ denotes the periodic convolution operator; **x**= [*x*(0),⋯,*x*(*N*−1)]; $\hat{x}=\;[\hat{x}(0),\cdots \;,\hat{x}(N-1)]$; **h**= [*h*(−*N*+1),⋯,*h*(0),⋯,*h*(*N*−1)], which is referred to as the convolution kernel.

From (5) or (6), if *h*(*l*)=*δ*(*l*), *l*∈ [−*N*+1,⋯,*N*−1], i.e.,

$$h(l)=\delta (l)=\left\{\begin{array}{l} 1,\;l=0,\\ 0,\;l\neq 0, \end{array}\right)$$

then $\hat{x}(n)=x(n)$. For example, it can be proven that if $\theta_{k}=\frac{2k\pi }{N}$ (the standard DFT), it results *h*(*l*)=*δ*(*l*), and $\hat{x}(n)=x(n)$. It also means the more *h*(*l*) is near *δ*(*l*), the more $\hat{x}(n)$ is near *x*(*n*).

**Figure 3:**
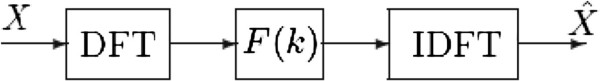
The flow chart of the proposed scheme to improve the reconstruction performance.

In order to improve reconstruction performance and avoid heavy calculation burden, an additional digital filter *F*(*k*) is inserted between DFT and IDFT, which is shown in Figure 3. According to (5) or (6), *F*(*k*) should be designed to drive the new convolution kernel $h(l)=\frac{1}{N}\sum_{k=0}^{N-1}e^{il\theta_{k}}F(k)$ to approach *δ*(*l*) as near as possible.

#### Remark 1

From (6), if *h*(*l*)≠*δ*(*l*), the aliasing distortion will be produced. The more *h*(*l*) is far from *δ*(*l*), the more aliasing distortion will be produced. *F*(*k*) acts as a correction term to make *h*(*l*) approach *δ*(*l*) as near as possible. However, the residual difference between *h*(*l*) and *δ*(*l*) not only depends on *F*(·) but also depends on the original kernel $\frac{1}{N}\sum_{k=0}^{N-1}e^{i\theta_{k}l}$. Generally, the difference can be reduced and cannot be removed. The farther the original kernel is different from *δ*(*l*), the more residual difference between *h*(*l*) and *δ*(*l*) may remain.

Obviously, the convolution kernel may be a complex number. In order to keep $\hat{x}(n)$ always as a real number, only the real part of *h*(*l*), $h_{r}(l)=\frac{1}{N}\sum_{k=0}^{N-1}\cos(\theta_{k}l)F(k)$, is retained in reconstructing the signal. The simplification can also reduce the calculation burden in the design *F*(*k*). Since it is unknown and may be very complicated, *F*(*k*) has to be expressed in the approximation models, such as Taylor series expansion,

(7) $$F(k)=\overset{S}{\underset{n=0}{\sum }}a_{n}k^{n},$$

where *S* is the degree of Taylor series; *a*_*n*_ denotes the coefficient to be estimated. The coefficient estimation has been achieved by a constrained optimization procedure in this paper. The requirement *h*_*r*_(0)=1 has been selected as the constrained condition, and $\sum_{l\neq 0}h_{r}^{2}(l)$ has been selected as the objective function. The constrained optimization problem becomes

(8) $$\begin{array}{ll} \underset{F(k)}{\min}\underset{l\neq 0}{\sum } & {\left(\overset{N-1}{\underset{k=0}{\sum }}\cos(\theta_{k}l)F(k)\right)}^{2},\\ \mathrm{s.t.}\;\;\;\frac{1}{N} & \overset{N-1}{\underset{k=0}{\sum }}F(k)-1=0. \end{array}$$

For the example in Figure 2, select *S*=18. The *fmincon* function in *Optimization Toolbox* of *Matlab* is employed in optimizing the nonlinear multivariable objective function (8). The results of optimization procedure, i.e. the coefficients of Taylor series (7) is shown Table 1.

**Table 1:** **The coefficients of Taylor series expansion of *F *( *k *)( *N *=16,*θ***_***k***_**=(2 *π k */ *N *)**^***0.8)***^

*a*_0_	0.001585600279081	*a*_9_	0.604131093429063
*a*_1_	-0.030192172552822	*a*_10_	0.108486066097029
*a*_2_	0.213462131921848	*a*_11_	-0.404865317972757
*a*_3_	-0.686735955366388	*a*_12_	-0.674935753170146
*a*_4_	1.122118847097341	*a*_13_	-0.298926140115388
*a*_5_	-0.387130280716676	*a*_14_	-0.084322132559712
*a*_6_	-0.834889525296690	*a*_15_	0.339643128448386
*a*_7_	-0.005116780492169	*a*_16_	0.662450316746720
*a*_8_	0.655962903880333	*a*_17_	0.239001712406937
*a*_18_	-0.529727742063988		

**Figure 4:**
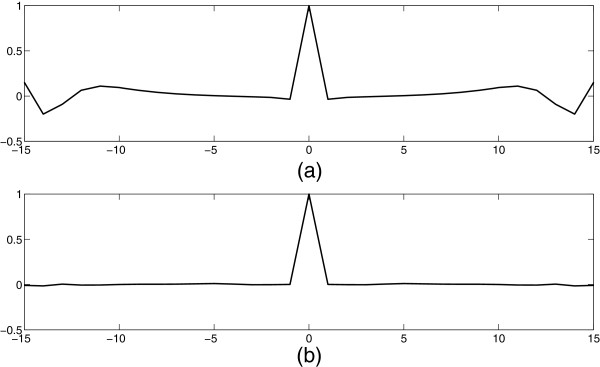
**The convolution vectors with and without the reconstruction filter (*N*=16,*θ***_***k***_**=(2*πk/N*)**^**0.8**^**).(a)** The convolution vector without the reconstruction filter, **(b)** with the reconstruction filter designed.

In the example, by inserting *F*(*k*), *h*_*r*_(*l*) is very near *δ*(*l*), which is shown in Figure 4 (b). However, without *F*(*k*), *h*_*r*_(*l*) is quite different from *δ*(*l*), which is shown in Figure 4 (a). The objective function in (8) can also be employed as an evaluation criterion. The result is $\sum_{l\neq 0}{\left(\frac{1}{16}\sum_{k=0}^{15}\cos(\theta_{k}l)F(k)\right)}^{2}=0.0013$. Without the filtering procedure, the evaluation criterion becomes $\sum_{l\neq 0}{\left(\frac{1}{16}\sum_{k=0}^{15}\cos(\theta_{k}l)\right)}^{2}$. It results $\sum_{l\neq 0}{\left(\frac{1}{16}\sum_{k=0}^{15}\cos(\theta_{k}l)\right)}^{2}=0.2093$. A much bigger value means the much more distortion will be brought into the reconstruction signal.

In this paper, *F*(*k*) is referred to as the reconstruction filter. It can improve the reconstruction performance significantly, which is illustrated by the same example above. The new reconstructed signal is plotted in Figure 2 (c), which is quite similar with the original signal.

The example illustrates the reconstruction performance can be improved by an additional digital filter designed properly. In the next section, a similar scheme is used in designing the reconstruction filter for CT reconstruction.

### The reconstruction filter for CT reconstruction

Let *f*(*n*,*m*) denote the discrete unknown image, $n,m\in \;[-\frac{N}{2},\cdots \;,\frac{N}{2}-1]$, *N* is a positive even integer. The image is projected on a detector from different scanning angles *θ*_*j*_,*j*∈[ 1,⋯*K*], which produces the projection data $p_{\theta_{j}}(l),l\in \;[-\frac{L}{2},\cdots \;,\frac{L}{2}-1]$, *L* is a positive even integer equals to or is larger than the maximum number of the discrete projection data at all directions. A special 2-D DFT of the image is defined as

(9) $$\begin{array}{l} S\left(k\cos\theta_{j},k\sin\theta_{j}\right)=S\left(k,\theta_{j}\right)\\ =\overset{\frac{N}{2}-1}{\underset{n=-\frac{N}{2}}{\sum }}\overset{\frac{N}{2}-1}{\underset{m=-\frac{N}{2}}{\sum }}f(n,m)\exp\left(-i2\pi k\frac{n\cos\theta_{j}+m\sin\theta_{j}}{L}\right), \end{array}$$

where $k\in \;[-\frac{L}{2},\cdots \;,\frac{L}{2}-1]$. *S*(*k* cos*θ*_*j*_,*k* sin*θ*_*j*_) can be approximately calculated using the following equation because of projection mechanism.

(10) $$S\left(k,\theta_{j}\right)\approx \overset{\frac{L}{2}-1}{\underset{l=-\frac{L}{2}}{\sum }}p_{\theta_{j}}(l)\exp\left(-i2\pi k\frac{l}{L}\right).$$

The reconstructed image $\hat{f}(n,m)$ can be obtained by the 2-D IDFT of *S*(*k*,*θ*_*j*_), which is

(11) $$\hat{f}(n,m)=\frac{1}{\text{LK}}\overset{K}{\underset{j=1}{\sum }}\overset{\frac{L}{2}-1}{\underset{k=-\frac{L}{2}}{\sum }}S\left(k,\theta_{j}\right)\exp\left(i2k\pi \frac{n\cos\theta_{j}+m\sin\theta_{j}}{L}\right).$$

By substituting (9) into (11), it results

(12) $$\begin{array}{ll} \hat{f}(n,m) & =\frac{1}{\text{LK}}\overset{K}{\underset{j=1}{\sum }}\overset{\frac{L}{2}-1}{\underset{k=-\frac{L}{2}}{\sum }}\overset{\frac{N}{2}-1}{\underset{n^{\prime }=-\frac{N}{2}}{\sum }}\overset{\frac{N}{2}-1}{\underset{m^{\prime }=-\frac{N}{2}}{\sum }}f(n^{\prime },m^{\prime })\\ \cdot \exp\left(i2\pi k\frac{\left(n-n^{\prime }\right)\cos\theta_{j}+\left(m-m^{\prime }\right)\sin\theta_{j}}{L}\right)\\ =\frac{1}{\text{LK}}\overset{\frac{N}{2}-1}{\underset{n^{\prime }=-\frac{N}{2}}{\sum }}\overset{\frac{N}{2}-1}{\underset{m^{\prime }=-\frac{N}{2}}{\sum }}f(n^{\prime },m^{\prime })\\ \cdot \left(\overset{K}{\underset{j=1}{\sum }}\overset{\frac{L}{2}-1}{\underset{k=-\frac{L}{2}}{\sum }}\exp(i2\pi k\frac{(n-n^{\prime })\cos\theta_{j}+(m-m^{\prime })\sin\theta_{j}}{L})\right)\\ =\overset{\frac{N}{2}-1}{\underset{n^{\prime }=-\frac{N}{2}}{\sum }}\overset{\frac{N}{2}-1}{\underset{m^{\prime }=-\frac{N}{2}}{\sum }}f\left(n^{\prime },m^{\prime }\right)h\left(n-n^{\prime },m-m^{\prime }\right). \end{array}$$

where

(13) $$\begin{array}{l} h\left(n-n^{\prime },m-m^{\prime }\right)\\ =\frac{1}{\text{LK}}\overset{K}{\underset{j=1}{\sum }}\overset{\frac{L}{2}-1}{\underset{k=-\frac{L}{2}}{\sum }}\exp\left(i2\pi k\frac{\left(n-n^{\prime }\right)\cos\theta_{j}+(m-m^{\prime })\sin\theta_{j}}{L}\right). \end{array}$$

Consider $n,n^{\prime },m,m^{\prime }\in [-\frac{N}{2},\cdots \;,\frac{N}{2}-1]$, *h*(*n*−*n*^′^,*m*−*m*^′^) can be regarded as an element of a matrix $H={\left\{h(n-n^{\prime },m-m^{\prime })\right\}}_{n,n^{\prime },m,m^{\prime }}$. The matrix, *H*∈*R*^(2*N*−1)×(2*N*−1)^, is named as the reconstruction matrix in this paper. The reconstruction procedure (12) can be expressed as following

(14) $$\hat{f}=f⊙H,$$

where ⊙ denotes the 2-D periodic convolution operator here; *H* is the 2-D convolution kernel.

**Figure 5:**
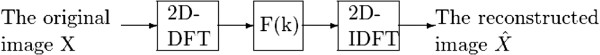
The flow Chart with the filtering process to improve the performance of CT reconstruction.

Generally, $\theta_{j+1}-\theta_{j}=\frac{\pi }{K}$ is a constant, i.e., the projection angles distribute equally in the region [0,*π*]. However, the frequency samples $\left(2\pi \frac{k}{L}\cos\theta_{j},2\pi \frac{k}{L}\sin\theta_{j}\right)$ do not distribute equally in the 2-D regions, such as [−*π*,*π*]^2^. Therefore, (9) and (11) form 2-D DFT and IDFT using the non-uniform frequency sampling, and the non-ignorable distortion will be brought into the reconstructed image. This problem is very similar with the 1-D example in the previous subsection. In order to reduce the distortion, an additional filter is necessary. It is similar with *F*(*k*) in Figure 3, and is shown in Figure 5. Further more, the idea behind the design of the additional filter for CT reconstruction is quite similar with that for 1-D signal reconstruction.

Similarly, the design has been achieved by a constrained optimization procedure. From (12) or (14), if *h*(*n*,*m*)=*δ*(*n*,*m*), $\hat{f}(n,m)=f(n,m)$. That is say, if *H* is a *δ* matrix (a matrix whose elements all are zeros except that the center element is one), the original image will be reconstructed exactly. It also means the more *h*(*n*,*m*) is close to *δ*(*n*,*m*), the more $\hat{f}(n,m)$ is close to *f*(*n*,*m*). Therefore, *F*(*k*) should be designed to drive *h*(*n*,*m*) to approach *δ*(*n*,*m*) as near as possible. Since it is unknown and is perhaps very complicated, *F*(*k*) has to be expressed in the approximation models, such as (7) or Fourier series expansion as following

(15) $$F(k)=a_{0}+\overset{M}{\underset{m=1}{\sum }}\left(a_{m}\sin\frac{\mathrm{mk\pi}}{N}+b_{m}\cos\frac{\mathrm{mk\pi}}{N}\right),$$

where *M* is the degree of Fourier series; *a*_*m*_ and *b*_*m*_ are the coefficients to be estimated.

With the additional filter *F*(*k*), the element of reconstruction kernel or (13) becomes

$$h\left(n,,,m\right)=\frac{1}{\text{LK}}\overset{K}{\underset{j=1}{\sum }}\overset{\frac{L}{2}-1}{\underset{k=-\frac{L}{2}}{\sum }}\exp\left(i2\pi \left(n\cos\theta_{j}+m\sin\theta_{j}\right)\frac{k}{L}\right)F\left(k\right).$$

Obviously, *h*(*n*,*m*) usually is a complex number. Similarly, only the real part of *h*(*n*,*m*), *h*_*r*_(*n*,*m*), is retained to ensure $\hat{f}(n,m)$ to be a real number. So, the Equation (13) has been simplified as following

$$h_{r}\left(n,,,m\right)=\frac{1}{\text{LK}}\overset{K}{\underset{j=1}{\sum }}\overset{\frac{L}{2}-1}{\underset{k=-\frac{L}{2}}{\sum }}\cos\left(2\pi \left(n\cos\theta_{j}+m\sin\theta_{j}\right)\frac{k}{L}\right)F\left(k\right).$$

In the design, the requirement *h*_*r*_(0,0)=1 (or $\frac{1}{\text{LK}}\overset{L-1}{\underset{k=0}{\sum }}F(k)=1$) is selected as the constrained condition, and $\underset{n\neq 0,m\neq 0}{\sum }h_{r}^{2}(n,m)$ is selected as the objective function. The constrained optimization problem becomes

(16) $$\begin{array}{ll} \underset{F(k)}{\min} & \underset{n\neq 0}{\sum }\underset{m\neq 0}{\sum }{\left(\overset{K}{\underset{j=1}{\sum }}\overset{\frac{L}{2}-1}{\underset{k=-\frac{L}{2}}{\sum }}\cos\left(2\pi \left(n\cos\theta_{j}+m\sin\theta_{j}\right)\frac{k}{L}\right)F\left(k\right)\right)}^{2},\\ \mathrm{s.t.}\;\; & \;\frac{1}{\text{LK}}\overset{L-1}{\underset{k=0}{\sum }}F(k)-1=0. \end{array}$$

#### Remark 2

From (16), it is obvious that *F*(*k*) depends on *N* (the image size) and *θ*_*j*_ (the scanning angles). The images of same size with same scanning process will have a same optimal *F*(*k*), vice versa. Generally, *F*(*k*) can reduce the difference between *h*_*r*_(*n*,*m*) and *δ*(*n*,*m*), however, it cannot remove the difference completely.

#### Remark 3

From (10), the projection and reconstruction model are relatively simple in this paper. There are many factors are not considered, such as the quantization error (the error that when a pixel is not at any projection line and has to be split between the two nearest projection lines) and projection noise. Even though for such a model, the optimization may be rather complicated and difficult. For example, the objective function is very complicated when the image size is large, and/or the number of projection angles is large.

## Results

### 

*Example 1.* In the example, the size of image is selected as *N*=256, the projection angles *θ*=[ 0^*o*^,3^*o*^,⋯,177^*o*^]. In the simulation, the expression of the objective function is very complicated, especially for the objective function. We used some tools in *Symbolic Math Toolbox* of *Matlab* in simplifying the procedure. The *fmincon* function in *Optimization Toolbox* of *Matlab* is used in finding the minimum of the objective function. The coefficients for the reconstruction filter *F*(*k*) in the form of (15) is shown in Table 2, in which *M*=9.

**Table 2:** **The coefficients of Fourier series expansion of *F *( *k *)( *N *= 256,*θ***_***k***_** = 0**^***o*, **^**3**^***o***^**,⋯,177**^***o***^**)**

*a*_0_	0.122730836679312		
*a*_1_	0.916483009109464	*b*_1_	-0.497982711366623
*a*_2_	0.462544582824757	*b*_2_	-0.120545376607102
*a*_3_	0.128736533815128	*b*_3_	0.040355538652453
*a*_4_	0.167851665809050	*b*_4_	-0.182518259771096
*a*_5_	0.315690517777738	*b*_5_	-0.094362761222605
*a*_6_	0.480769918991562	*b*_6_	0.285895915083407
*a*_7_	-0.189184291984498	*b*_7_	0.563061220832784
*a*_8_	-0.335163337055193	*b*_8_	-0.086997019632140
*a*_9_	0.023005162904716	*b*_9_	-0.087383132662148

**Figure 6:**
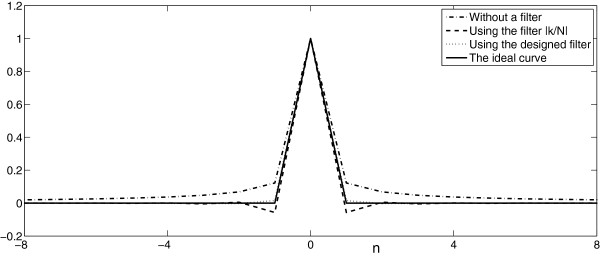
**The comparison of the 0-*th* rows of reconstruction matrixes with and without the reconstruction filters (*N*=256,*θ*=[0**^***o***^***,*3**^***o***^**,⋯,177**^***o***^**]).**

The reconstruction filter can drive the reconstruction kernel *h*_*r*_(*n*,*m*) to be very close to *δ*(*n*,*m*), which can be illustrated in Figure 6. In the Figure, the dot curve is the center row (0-*th* row, or *h*_*r*_(0,*m*)) of *h*_*r*_(*n*,*m*) using the reconstruction filter designed, while the dash curve is the corresponding row of *h*_*r*_(*n*,*m*) using the ramp filter |*k*/*N*|. The dash-dot curve is the corresponding row without a filter, and the solid curve denotes the ideal curve *δ*(*l*). It shows the curve using the designed filter is closest to the ideal curve *δ*(*l*). Consider the symmetry of the reconstruction matrix, it implies the designed filter can make *h*_*r*_(*n*,*m*) be more close to *δ*(*n*,*m*) than the ramp filter.

**Figure 7:**
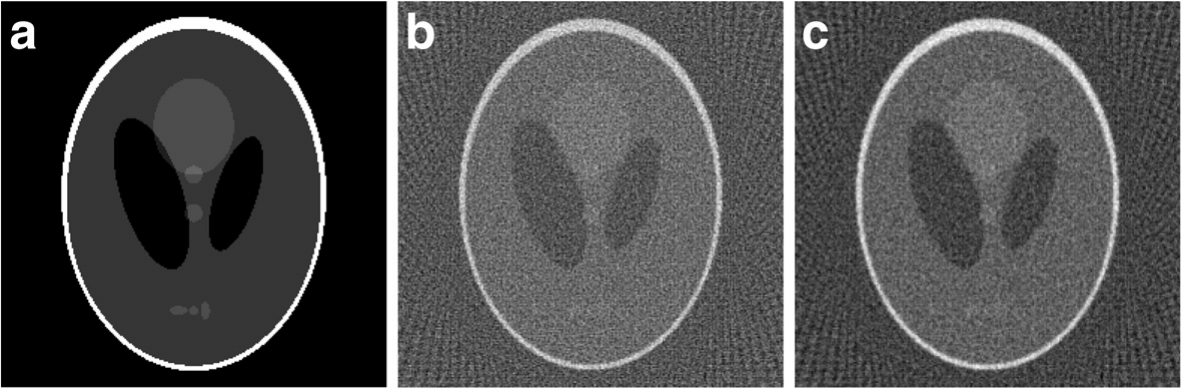
**The original image and the reconstructed images using FBP algorithm with different reconstruction filters (*N*=256,*θ*=[0**^***o***^**,3**^***o***^**,⋯,177**^***o***^**]).(a)** The original image, **(b)** using the ramp filter |*k*/*N*|, **(c)** using the designed filter.

A simulation example is employed to illustrate the availability of the reconstruction filter designed. The original image is of 256×256 pixels, *head phantom*, which is shown in Figure 7 (a). The projections are calculated using *radon* function in *Matlab* with the rotation angle interval 3^*o*^. Since the noise in the projection data are usually modeled by the Poisson distribution [17,18], we suppose the projection data is polluted by Poisson noise whose mean is 5 (also the variance is 5, while the maximum of projection data is 66). It is then reconstructed using FBP algorithm with different reconstruction filters, which are shown in Figure 7 (b) and (c). It shows the small white circle in Figure (c) has more obvious boundary than that in Figure (b).

Three criteria, *MSE* (Mean Square Error), *UQI* (Universal Quality Index) and *MI* (mutual information), are employed to assess the efficiency of the designed filter, which are defined as following [19].

$$\text{MSE}\left(f^{i},f^{0}\right)=\frac{1}{M}\sqrt{\overset{M-1}{\underset{k=0}{\sum }}{\left(f_{k}^{i}-f_{k}^{0}\right)}^{2}},$$

where $f_{k}^{i}$ and $f_{k}^{0}$ denote the pixels of the reconstructed image *f*^*i*^ and reference image *f*^0^, respectively; *M* is the total number of pixels in the selected region. Since it is a simulation example, the original image is known and selected as the reference image. *UQI* is defined as following

$$\text{UQI}\left(f^{i},f^{0}\right)=\frac{2\text{Cov}\{f^{i},f^{0}\}}{\sigma_{i}^{2}+\sigma_{0}^{2}}\frac{2{\bar{f}}_{i}{\bar{f}}_{0}}{{\bar{f}}_{i}^{2}{\bar{f}}_{0}^{2}},$$

where ${\bar{f}}_{i}$ and $\sigma_{i}^{2}$ denote the image mean and variance, respectively; *Cov*{*f*^*i*^,*f*^0^} denote the covariance of the reconstructed image *f*^*i*^ and reference image *f*^0^. The mean, variance and covariance are defined as the following

$$\begin{array}{l} {\bar{f}}^{i}=\frac{1}{M}\overset{M-1}{\underset{k=0}{\sum }}f_{k}^{i},\\ \sigma_{i}^{2}=\frac{1}{M-1}\overset{M-1}{\underset{k=0}{\sum }}{\left(f_{k}^{i}-{\bar{f}}_{i}\right)}^{2},\\ \text{Cov}\{f^{i},f^{0}\}=\frac{1}{M-1}\overset{M-1}{\underset{k=0}{\sum }}\left(f_{k}^{i}-{\bar{f}}_{i}\right)\left(f_{k}^{0}-{\bar{f}}_{0}\right). \end{array}$$

*UQI* measures the pixel-to-pixel similarity between the reconstructed *f*^*i*^ and reference image *f*^0^. It was designed by modeling any image distortion as a combination of three factors: loss of correlation, luminance distortion, and contrast distortion. Its value ranges between 0 and 1. The closer to 1 the *UQI* value is, the more similar to the reference image the reconstructed image is.

When the reconstructed image and reference image are interpreted as “stochastic processes”, *MI* is used for measuring their mutual dependence.

$$\text{MI}\left(f^{i},,,f^{0}\right)=\overset{N^{\prime }-1}{\underset{k=0}{\sum }}\overset{N^{\prime }-1}{\underset{n=0}{\sum }}p\left(f_{k}^{i},,,f_{k}^{0}\right)\log\left(\frac{p(\overset{i}{\underset{k}{f}},f_{k}^{0})}{p(\overset{i}{\underset{k}{f}})p(f_{k}^{0})}\right),$$

where $p(f_{k}^{i})$ and $p(f_{k}^{0})$ denote the marginal densities of *f*^*i*^ and *f*^0^, respectively, which are calculated using the corresponding histograms; the joint density $p(f_{k}^{i},f_{k}^{0})$ is estimated from the joint histogram of *f*^*i*^ and *f*^0^; *N*^′^ denotes the number of bins in the histogram. *UQI* measures the pixel-to-pixel dependence of the reconstructed image on the reference image, MI measures the histogram correlation between them. *MI* can be highly sensitive to small differences between visually similar images.

The results for the example in the form of the three criteria are showed in Table 3. The results illustrate the designed filters have better reconstruction performance than the standard ramp filter.

**Table 3:** **The results of performance evaluation in Example **1

**Criteria**	**MSE**	**UQI**	**MI**
The ramp filter	1.237E-3	0.3429	0.2758
The designed filter	9.129E-4	0.4751	0.4563

### 

*Example 2.* In one example, the size of image is *N*=128×128, *θ*= [ 0^*o*^,4^*o*^,⋯,176^*o*^], the coefficients for *F*(*k*) in the form of (15) is listed in Table 4. In another example, the size of image is *N*=256×256, *θ*= [ 0^*o*^,1^*o*^,⋯,179^*o*^], the coefficients for *F*(*k*) in the form of (15) is listed in Table 5.

**Table 4:** **The coefficients of Fourier series expansion of *F *( *k *) ( *N *= 128,*θ***_***k***_**=4*k***^***o***^**, *k * = 0,⋯,44)**

*a*_0_	0.167768278296600		
*a*_1_	0.742071367715883	*b*_1_	-0.050516729362635
*a*_2_	-0.007222808863187	*b*_2_	-0.257039580419913
*a*_3_	0.205121481459339	*b*_3_	-0.107883292543924
*a*_4_	0.080764672022586	*b*_4_	0.021273467611896
*a*_5_	-0.013240204304737	*b*_5_	-0.106948545272204
*a*_6_	0.297619345807562	*b*_6_	-0.114131247626016
*a*_7_	0.184808294809679	*b*_7_	0.324654368672888
*a*_8_	-0.198785426563876	*b*_8_	0.153168707602131
*a*_9_	-0.059263999435747	*b*_9_	-0.059403297538579

**Table 5:** **The coefficients of Fourier series expansion of *F *( *k *) ( *N * = 256,*θ***_***k***_**=*k ***^***o***^**, *k * = 0,⋯,179)**

*a*_0_	0.209555620787293		
*a*_1_	0.693546995671764	*b*_1_	-0.201010002024803
*a*_2_	0.099842536865856	*b*_2_	-0.101104690137033
*a*_3_	-0.152784751604807	*b*_3_	-0.180901677852717
*a*_4_	0.248221333497748	*b*_4_	-0.353671098582667
*a*_5_	0.308013835831055	*b*_5_	-0.032248278459190
*a*_6_	0.377740474429379	*b*_6_	0.104300291184828
*a*_7_	0.075385012026711	*b*_7_	0.465974969229510
*a*_8_	-0.294404584616438	*b*_8_	0.114991610901604
*a*_9_	-0.056064644119820	*b*_9_	-0.088997280339292

**Figure 8:**
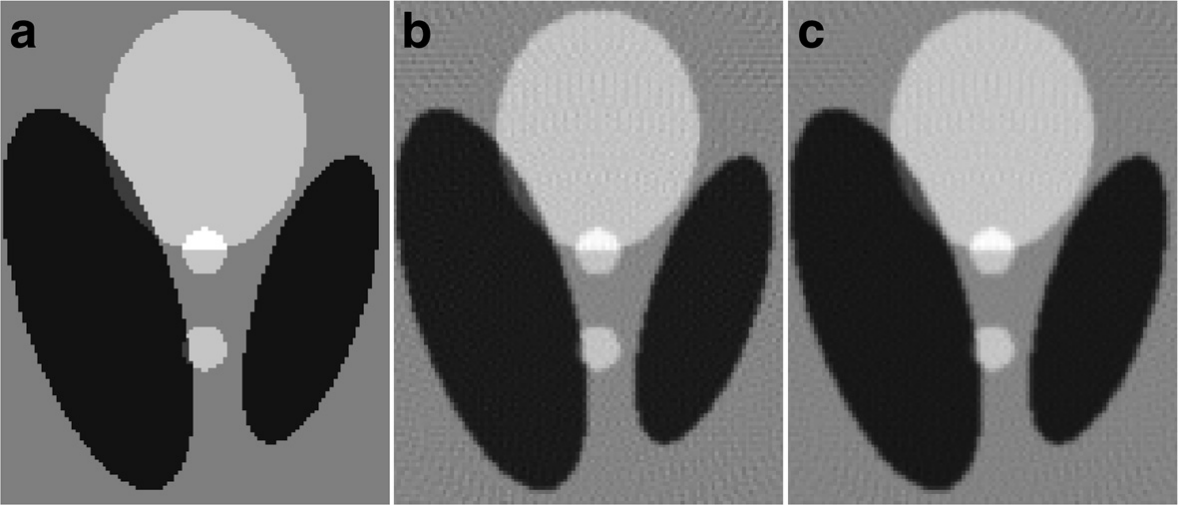
**The small regions of original image and the reconstructed images using FBP algorithm with different reconstruction filters ( *N *=256, θ=[0**^***o***^**,1**^***o***^**,⋯,179**^***o***^**]).(a)** The original image, **(b)** using general filter |*k*/*N*|**(c)** using the designed filter.

Two similar simulation examples are employed to illustrate the availability of the designed filters. They are the *head phantom* of sizes 128×128 and 256×256 pixels, respectively. The projections are calculated using *radon* function in *Matlab* with the rotation angle interval 4^*o*^ and 1^*o*^, respectively. The image is reconstructed using FBP algorithm with different reconstruction filters. The results of performance evaluation in the form of *MSE*, *UQI* and *MI* are showed in Table 6. The identical small regions of the original image and reconstructed images for the latter example are shown in Figure 8. It shows the artifact has been reduced more efficiently using the designed filter at the interior of white circle and other regions. The results illustrate the designed filters have better performance than the ramp filter for CT reconstruction.

**Table 6:** The results of performance evaluation in Example 2

**(Case 1: *S i z e *=128∗128,*θ***_***k***_**=4*k***^***o***^**, *k *=0,⋯,44)**			
**Criteria**	**MSE**	**UQI**	**MI**
The ramp filter	1.399E-3	0.6454	0.8022
The designed filter	1.229E-3	0.6914	0.8518
**(Case 2: *S i z e *=256∗256,*θ***_***k***_**=*k***^***o***^**, *k *=0,⋯,179)**			
**Criteria**	**MSE**	**UQI**	**MI**
The ramp filter	2.645E-4	0.9219	0.9289
The designed filter	2.415E-4	0.9384	0.9301

*Summary*: The simulation examples demonstrate that *F*(*k*) depends on the image size and the projection angles. The images of different sizes and/or with different projection angles usually have different optimal *F*(*k*), which makes the problem rather complicated. In order to simplify the expression of reconstruction filter, a much more simple way is to substitute the expression (15) by fitting the filters designed. It is a hyperbolic function as following

$$F\left(k\right)=\frac{\exp(a\sin(\pi k/N))-\exp(-a\sin(\pi k/N))}{\exp(a\sin(\pi k/N))+\exp(-a\sin(\pi k/N))}$$

where *a* is a parameter selected in region [ 0.5,2]. For example, *a*=1.65 for the *Example*1, and *a*=1 and *a*=0.6 for the filters in *Example*2, respectively.

Generally, the more complicated model *F*(*k*) is of, the better properties it has. However, a very much complicated model *F*(*k*) means very much heavy burden of calculation, which may cause the optimal parameters even the valid parameters cannot be found. On the other hand, the difference between *h*_*r*_(*n*,*m*) and *δ*(*n*,*m*) can only be reduced and cannot be removed. So a moderate complicated model *F*(*k*), such as (7) with *S*=18 and (15) with *M*=9, is an appropriate choice.

## Conclusion

For the continuous image model and scanning, FBP algorithm is perfect in the mathematics principle, in which the ramp filter |*ω*| (it is named as the reconstruction filter in this paper) plays an important role. However, it necessarily causes degradation in the reconstructed images when the continuous image model is discretized and the continuous scanning is substituted by the discrete (finite) scanning in the practical calculation. It means the standard discrete version of ramp filter, |*k*/*N*|, is not the optimal for the discrete FBP algorithm. In this paper, a novel scheme is proposed to design the new reconstruction filters to substitute the ramp filter. According to analysis, the reconstructed image can be regarded as the 2-D IDFT of 2-D DFT of the original image using non-uniform frequency sampling. It is also equivalent to a 2-D periodic convolution of the original image and a special 2-D kernel (it is named as the reconstruction matrix in this paper). The more the reconstruction matrix is close to a *δ*-matrix (a matrix whose elements all are zeros except the center element is one), the more the reconstructed image is close to the original image. Therefore, the reconstruction filters are designed to drive the reconstruction matrixes approach *δ*-matrix as near as possible. The designs are achieved by the constrained optimization procedures. Some simulation examples have been finished. The results demonstrate the filters designed can make the reconstruction matrixes more close to *δ*-matrix than the ramp filter. The performance evaluation of CT reconstruction also shows the designed filters have outstanding superiority over the ramp filter in the term of three general criteria, such as MSE, UQI and MI.

## Competing interests

The authors declare that they have no competing interests.

## Authors’ contributions

Shi carried out all the mathematics analysis, design, simulation, and draft the manuscript. Luo carried out the revision of the spelling, wording, and expression and given some important advices on the framework of the manuscript. Both authors read and approved the final manuscript.
